# Association of Time-Varying Blood Pressure With Chronic Kidney Disease Progression in Children

**DOI:** 10.1001/jamanetworkopen.2019.21213

**Published:** 2020-02-14

**Authors:** Ben Christopher Reynolds, Jennifer Lynn Roem, Derek Kai Sing Ng, Mina Matsuda-Abedini, Joseph Thomas Flynn, Susan Lynn Furth, Bradley Alan Warady, Rulan Savita Parekh

**Affiliations:** 1 Department of Paediatric Nephrology, Royal Hospital for Children, Glasgow, United Kingdom; 2 Department of Epidemiology, Johns Hopkins Bloomberg School of Public Health, Baltimore, Maryland; 3 Division of Nephrology, Department of Pediatrics, Hospital for Sick Children, University of Toronto, Toronto, Ontario, Canada; 4 Department of Pediatrics, University of Washington, Seattle; 5 Division of Nephrology, Seattle Children’s Hospital, Seattle, Washington; 6 Division of Nephrology, The Children’s Hospital of Philadelphia, Philadelphia, Pennsylvania; 7 Division of Pediatric Nephrology, Children’s Mercy Kansas City, Kansas City, Missouri; 8 Division of Nephrology, Department of Pediatrics and Medicine, Hospital for Sick Children, University Health Network, University of Toronto, Toronto, Ontario, Canada

## Abstract

**Importance:**

Optimal blood pressure (BP) management in children with chronic kidney disease (CKD) slows progression to end-stage renal disease. Studies often base progression risk on a single baseline BP measurement, which may underestimate risk.

**Objective:**

To determine whether time-varying BP measurements are associated with a higher risk of progression of CKD than baseline BP measurements.

**Design, Setting, and Participants:**

The ongoing longitudinal, prospective cohort study Chronic Kidney Disease in Children (CKID) recruited children from January 19, 2005, through March 19, 2014, from pediatric nephrology centers across North America, with data collected at annual study visits. Participants included children aged 1 to 16 years with a diagnosis of CKD and a glomerular filtration rate (GFR) of 30 to 90 mL/min/1.73 m^2^. Data were analyzed from February 11, 2005, through February 13, 2018.

**Exposures:**

Office BP measurement classified as less than 50th percentile, 50th to less than 90th percentile, or at least 90th percentile. Blood pressure categories were treated as time fixed (baseline) or time varying (updated at each visit) in models.

**Main Outcomes and Measures:**

A composite renal outcome (50% GFR reduction from baseline, estimated GFR less than 15 mL/min/1.73 m^2^, or dialysis or transplant). Pooled logistic models using inverse probability weighting estimated the hazard odds ratio (HOR) of the composite outcome associated with each BP category stratified by CKD diagnosis.

**Results:**

A total of 844 children (524 [62.1%] male; median age, 11 [interquartile range, 8-15] years; 151 [17.9%] black; 580 [68.7%] with nonglomerular CKD; and 264 [31.3%] with glomerular CKD) with complete baseline data and median follow-up of 4 (interquartile range, 2-6) years were included. One hundred ninety-six participants with nonglomerular diagnoses (33.8%) and 99 with glomerular diagnoses (37.5%) reached the composite outcome. Baseline systolic BP in at least the 90th percentile was associated with a higher risk of the composite outcome (HOR for nonglomerular disease, 1.58 [95% CI, 1.07-2.32]; HOR for glomerular disease, 2.85 [95% CI, 1.64-4.94]) compared with baseline systolic BP in less than the 50th percentile. Time-fixed estimates were substantially lower compared with time-varying systolic BP percentile categories (HOR among those with nonglomerular CKD, 3.75 [95% CI, 2.53-5.57]; HOR among those with glomerular diagnoses, 5.96 [95% CI, 3.37-10.54]) comparing those at or above the 90th percentile vs below the 50th percentile. Adjusted models (adjusted for proteinuria and use of antihypertensives) attenuated the risk in nonglomerular CKD (adjusted HOR for baseline measurement, 1.52 [95% CI, 0.98-2.36]; adjusted HOR for time-varying measurement, 2.25 [95% CI, 1.36-3.72]) and in glomerular CKD (adjusted HOR for baseline, 0.97 [95% CI, 0.39-2.36]; adjusted HOR for time-varying measurement, 1.41 [95% CI, 0.65-3.03]). Similar results were observed for diastolic BP.

**Conclusions and Relevance:**

Among children with nonglomerular CKD included in this study, elevated time-varying BP measurements were associated with a greater risk of CKD progression compared with baseline BP measurement. This finding suggests that previous studies using only baseline BP likely underestimated the association between BP and CKD progression.

## Key Points

**Question:**

Are elevated longitudinal (time-varying) blood pressure measurements associated with a greater risk of disease progression when compared with baseline (time-fixed) blood pressure measurements in children with chronic kidney disease?

**Findings:**

In this cohort study of 844 children with chronic kidney disease, those with baseline elevated systolic or diastolic blood pressure were at greater risk of disease progression. Elevated time-varying systolic and diastolic blood pressures were associated with increased risk among children with glomerular disease, even after adjustment for proteinuria and use of antihypertensives, and were more strongly associated with kidney disease progression than baseline blood pressure.

**Meaning:**

These findings suggest that risk estimated using time-fixed blood pressure alone may underestimate the importance of updated blood pressure measurements in the assessment of chronic kidney disease progression.

## Teaser

This cohort study assesses whether time-varying vs baseline blood pressure measurements are associated with a higher risk of disease progression among children with chronic kidney disease.

## Introduction

Hypertension is a common comorbidity in children with CKD^1,2^ and is associated with progression of CKD in cohort studies and trials.^3,4,5^ Effective treatment of hypertension is known to slow the rate of progression to end-stage renal disease, as highlighted by the Effect of Strict Blood Pressure Control and Angiotensin-Converting Enzyme Inhibition on the Progression of Chronic Renal Failure in Pediatric Patients (ESCAPE) trial,^4^ which demonstrated that improved blood pressure (BP) management delayed the progression of CKD, especially among those with proteinuria. Blood pressure control in children with CKD may require several medications to optimize BP, and the most commonly used classes are angiotensin-converting enzyme inhibitors and angiotensin receptor blockers (ACEis/ARBs) or calcium channel blockers.^4^

Office BP measurements are commonly used in clinical practice, providing an immediate, noninvasive assessment during clinical review, and clinicians often consider a patient’s BP history, whether home monitoring or during clinic visits, when making treatment decisions. These time-varying BP measurements may reflect a change in a patient’s clinical status (ie, a child with previous normotension may develop hypertension and vice versa). However, clinical studies assessing risk often use baseline BP at study entry—typically an office BP measurement—and this variable is treated as a time-fixed value. For example, the Chronic Kidney Disease in Children (CKID) and ESCAPE studies both demonstrated a similar association of elevated baseline systolic BP and progression in children.^6,7,8^ Incorporating time-varying BP would reflect clinical practice and ongoing antihypertensive management better than using a single measurement, but analysis should also account for other time-varying potential confounders. The availability of additional clinical data ensured by updating the BP record over time likely provides more valid estimates to assess risk than a single time-fixed BP record alone. Several studies have demonstrated that longitudinal measurements of BP in adults were more strongly associated with progression of CKD than baseline BP measurements.^9,10,11,12^ These results may reflect ongoing BP management and whether treated BP is well controlled, which are important factors in CKD management.

Among children in the CKID study, those with nonglomerular diagnoses (typically characterized by congenital anomalies of the urinary tract or other obstructive disorders) tend to experience progression more slowly than children with glomerular diseases (including focal segmental glomerulosclerosis, hemolytic uremic syndrome, and systemic immunological disease diagnoses).^13^ Compared with children with nonglomerular diagnoses, those with glomerular diseases also have a higher burden of nephrotic range proteinuria and higher BP.^6,8,14^ The combination of proteinuria and time-varying BPs may confer a high risk of progression that is reflected in the rapid immediate decline.

Among pediatric hypertension studies in CKD, the extent to which longitudinal BP measurements are more informative than baseline office BP measurements in assessing the risk of disease progression is less established. We hypothesized that longitudinal, time-varying measures of systolic and diastolic BP would be more strongly associated with CKD progression over time compared with baseline (time-fixed) BP measures alone stratified by CKD cause.

## Methods

### Study Population

Data were obtained from participants enrolled in the CKID study, a prospective cohort study ongoing in 54 North American pediatric nephrology centers, recruited children from January 19, 2005, through March 19, 2014, with data collected at annual visits.^15^ The study design and ongoing conduct were approved by an independent external advisory committee established by the National Institutes of Health, and ethics approval was granted at each participating center. Written informed consent was obtained from parents, and patient assent was obtained when age appropriate. The study followed the Strengthening the Reporting of Observational Studies in Epidemiology (STROBE) reporting guideline.

Detailed methods were previously published.^15^ Briefly, children aged 1 to 16 years with a glomerular filtration rate (GFR) of 30 to 90 mL/min/1.73 m^2^ and a CKD diagnosis at enrollment were eligible to participate. Exclusion criteria included solid organ, bone marrow, or stem cell transplant; requirement for renal replacement therapy in the 3 months before enrollment; treatment for cancer, leukemia, or HIV; and pregnancy. Annual study visits collected data on CKD severity, cardiovascular health, growth, therapies, and neurocognitive functioning.

### Exposure

Office BP was measured as previously described^2^ after a minimum rest period of 5 minutes in the sitting position, using a calibrated aneroid sphygmomanometer on the right arm with manual auscultation of the brachial artery by trained study coordinators at each annual study visit. Blood pressure measurements from additional clinical visits were not used. Systolic BP was defined as appearance of the first Korotkoff sound; diastolic BP, the fifth Korotkoff sound. Three BP measurements were obtained at 30-second intervals, and the mean was calculated. The purpose of this approach was to reduce the variability (measurement error) of a single measurement. Blood pressure measurements were converted to percentiles according to the normative population data, adjusting for age, sex, and height.^16^ Baseline BP percentile was defined as the first available measurement in the study. Time-varying BP percentiles were updated at each available study visit so that all longitudinal study visit data were incorporated. Baseline and time-varying BP measurements were categorized into 3 groups defined as less than the 50th percentile (reference group), 50th to less than 90th percentile, or 90th percentile or greater. For time-varying BP percentiles, each participant was classified according to their BP measurement at each annual visit and was considered in that category until a new BP measurement was obtained. Records in which BP was not obtained were not included in the analysis.

### Outcome

The primary outcome was a composite renal outcome, defined as the earliest of 3 events: initiation of renal replacement therapy (dialysis or transplant), estimated GFR (eGFR) of less than 15 mL/min/1.73 m^2^, or 50% eGFR reduction from baseline. For this analysis, eGFR measured at each annual visit was used, based on the modified Schwartz equation using height and levels of serum creatinine, cystatin C, and blood urea nitrogen.^17^

### Covariates

Covariates identified as potential confounders included age, sex, black race, antihypertensive use (defined as ACEi or ARB use vs non-ACEi/ARB use [most commonly calcium channel blockers]), immunosuppressant use (participants with glomerular disease only), eGFR (as above), and age- and sex-specific *z* score for body mass index (BMI; calculated as weight in kilograms divided by height in meters squared). Because proteinuria is closely linked to elevated BP^6,8,14^ and the causal directionality between proteinuria and BP is unclear (ie, proteinuria may be considered a mediator of the BP to composite renal outcome association), models were included that adjusted for the aforementioned covariates with and without proteinuria (as a continuous variable in the logarithm scale). For baseline BP models, only baseline covariates were included for adjustment. For time-varying BP models, adjustment occurred for baseline and time-updated covariates as well as clinical history of selected variables, including BP *z* score, eGFR, BMI *z* score, antihypertensive therapy, and proteinuria, from 2 previous visits, if available.

### Statistical Analysis

Data were analyzed from February 11, 2005, through February 13, 2018. The primary goal was to compare the association between BP and development of the composite outcome, when BP is treated as a baseline or time-varying exposure. Because exposures were measured at regular annual intervals, we used a discrete time survival analysis.^18^ Pooled logistic regression models were used to quantify the association between BP categories (time-fixed or time-varying for systolic and diastolic BPs) and the risk of the composite outcome during follow-up visits.

Coefficients for BP percentile groups from these models are interpreted as hazard odds ratios (HORs) and were assumed to be time constant. Generalized estimating equations were used to account for repeated measurements within individuals.

To comprehensively describe the association between BP (baseline or time-varying for systolic and diastolic) and the outcome for nonglomerular and glomerular CKD, 3 models were fit. Model 1 was unadjusted, model 2 was adjusted for the covariates listed earlier but not proteinuria, and model 3 was additionally adjusted for proteinuria. The HORs were interpreted as the relative hazard for a hypothetical group of individuals with persistent higher BP percentile group levels compared with the hazard among those who remained in the group with BP of less than the 50th percentile.

Weighting methods were used to account for confounding using similar methods previously described.^12^ For adjusted time-fixed BP models, single inverse probability of exposure weights (IPEWs) were incorporated based on baseline covariates only.^19^ For adjusted time-varying BP models, weights were based on IPEWs that included time-updated covariates and inverse probability of censoring weights (IPCWs) to account for potential informative dropout. The final inverse probability weights were calculated as the product of IPEW and IPCW at each point.

For time-fixed and time-varying BP measurements, IPEWs were calculated using a multinomial logistic regression to estimate the probability of observed BP category conditional on covariates. In the time-varying models, IPCWs were estimated from a regular logistic regression model in which the probability of censoring was modeled as a dropout at the next visit, conditional on covariates. All weights were stabilized according to the marginal probability of the observed exposure. Time-varying weights were truncated based on the distribution of the final weights such that weights below the 1st percentile and above the 99th percentile were set to their respective percentile, which were approximately 0.20 and 4.00, respectively. The overall proportion of person-visits that required truncation was low, at 2% for nonglomerular or glomerular groups.

All analyses were completed for systolic and diastolic BP percentile categories and stratified by diagnosis as nonglomerular or glomerular CKD. For baseline comparisons, the Wilcoxon rank sum test and the Cochran-Armitage test compared differences in continuous and binary variables across BP percentile categories, respectively. Hazard odds ratios, 95% CIs, and *P* values from the overall type 3 tests for trends were reported. Models for time-varying BP are interpreted as the risk of the composite renal outcome for a hypothetical individual with BP across study visits that was consistently in that BP category. Statistical analysis was performed using SAS, version 9.4 (SAS Institute Inc). Two-sided *P* < .05 indicated statistical significance.

## Results

A total of 891 children were enrolled in the CKID study and contributed 5077 individual visits. Of these, 447 visits (8.8%) were omitted from the analysis owing to incomplete BP measurements. A further 389 visits (7.7%) had insufficient clinical data, specifically proteinuria, BMI, and GFR, leaving 4241 visits available for analysis. Complete baseline BP and covariate data were available for 844 participants (580 [68.7%] with nonglomerular CKD and 264 [31.3%] with glomerular CKD) (524 [62.1%] male and 320 [37.9%] female; median age, 11 [interquartile range {IQR}, 8-15] years; 151 [17.9%] black). Where BMI, proteinuria, or GFR data were missing from later visits (427 [10.1%]), the previous measurement was carried forward for as many as 1 visit in 256 visits (6.0%). Participants with nonglomerular CKD contributed a total of 3195 person-visits, and 196 of 580 participants (33.8%) reached the composite renal outcome. Participants with glomerular CKD contributed 1046 person-visits, and 99 of 264 (37.5%) developed the composite renal outcome. A full characterization of visits is provided in eTable 1 in the Supplement, and eTable 2 in the Supplement enumerates the type of event that defined composite renal outcome.

Loss to follow-up for reasons other than the composite event affected 93 of the 844 participants (11.0%). The remaining participants reached the composite event (295 [35.0%]) or were still active in the study (456 [54.0%]) and contributed a median of 4 (IQR, 2-6) years of follow-up. Of the 93 participants who were disenrolled, 7 had died and the rest had left the study owing to aging out of care at pediatric centers. No participants left the study owing to a severe kidney or cardiovascular complication that precluded observing a renal replacement therapy event.

Baseline characteristics associated with systolic and diastolic BPs in the less than 50th, 50th to less than 90th, and 90th or greater percentiles are shown for participants with nonglomerular CKD (Table 1) and glomerular CKD (Table 2). Among children with nonglomerular CKD (Table 1), systolic BP of at least the 90th percentile was associated with younger age (median, 8 [IQR, 6-11] years), black race (23 of 122 [18.9%]), lower height percentiles (median, 20th [IQR, 5th-47th] percentile), and higher diastolic BP (median, 90th [IQR, 70th-97th] percentile). Elevated diastolic BP of at least the 90th percentile was associated with younger age (median, 9 [IQR, 5-3] years) and black race (22 of 116 [19.0%]) as well as male sex (89 of 116 [76.7%]) and higher systolic BP (median, 90th [IQR, 77th-97th] percentile). Higher diastolic BP percentiles were also associated with less use of antihypertensives (61 of 116 [52.6%]) and ACEis/ARBs (48 of 116 [41.4%]). Forty of 258 participants (15.5%) had systolic BP in the 50th to less than 90th percentiles, and 25 of 122 (20.5%) had systolic BP of at least the 90th percentile at all of their study visits; 53 of 302 (17.5%) and 14 of 116 (12.1%), respectively, remained in those same diastolic BP categories. In the reference groups (<50th percentile for systolic and diastolic BP), 78 of 200 (39.0%) for systolic BP and 41 of 162 (25.3%) for diastolic BP remained. Overall, 143 (24.7%) and 108 (18.6%) of the 580 participants in the nonglomerular disease were consistently in 1 of the 3 systolic and diastolic BP groups, respectively.

**Table 1.** Demographic and Clinical Characteristics by Baseline SBP and DBP Percentiles for 580 Participants With Nonglomerular Disease

Baseline Characteristic	Baseline SBP Percentile				Baseline DBP Percentile			
	<50th (n = 200)	50th to <90th (n = 258)	≥90th (n = 122)	*P* Value^a^	<50th (n = 162)	50th to <90th (n = 302)	≥90th (n = 116)	*P* Value^a^
Age, median (IQR), y	12 (9-15)	10 (7-13)	8 (6-11)	<.001	11 (8-15)	10 (7-13)	9 (5-13)	.001
Male, No. (%)	128 (64.0)	175 (67.8)	81 (66.4)	.58	95 (58.6)	200 (66.2)	89 (76.7)	.002
Black race, No. (%)	19 (9.5)	39 (15.1)	23 (18.9)	.02	14 (8.6)	44 (14.6)	22 (19.0)	.01
Height percentile, median (IQR)	38 (11-60)	27 (7-56)	20 (5-47)	.01	24 (8-56)	31 (8-60)	22 (8-48)	.27
BMI percentile, median (IQR)	56 (29-87)	62 (33-89)	68 (36-90)	.16	62 (37-86)	61 (33-90)	63 (33-89)	.99
≥85th Percentile for BMI, No. (%)	56 (28.0)	75 (29.1)	39 (32.0)	.47	45 (27.8)	91 (30.1)	34 (29.3)	.74
GFR, median (IQR), mL/min/1.73 m^2^	49 (37-62)	50 (38-62)	49 (36-60)	.78	47 (35-58)	51 (38-64)	49 (38-60)	.05
Ratio of urine protein to creatinine levels, median (IQR), mg/mg	0.26 (0.10-0.78)	0.29 (0.12-0.79)	0.31 (0.11-0.82)	.41	0.27 (0.11-0.73)	0.27 (0.10-0.80)	0.32 (0.12-0.84)	.67
Nephrotic-range proteinuria, No. (%)^b^	17 (8.5)	21 (8.1)	9 (7.4)	.73	11 (6.8)	26 (8.6)	10 (8.6)	.55
Matched BP percentile, median (IQR)^c^	54 (35-73)	74 (53-87)	90 (70-97)	<.001	40 (15-69)	65 (42-83)	90 (77-97)	<.001
Antihypertensive use, No. (%)	113 (56.5)	132 (51.2)	72 (59.0)	.85	111 (68.5)	145 (48.0)	61 (52.6)	.003
ACEi/ARB use, No. (%)	100 (50.0)	110 (42.6)	56 (45.9)	.35	99 (61.1)	119 (39.4)	48 (41.4)	<.001

Abbreviations: ACEi, angiotensin converting enzyme inhibitor; ARB, angiotensin receptor blocker; BMI, body mass index (calculated as weight in kilograms divided by height in meters squared); DBP, diastolic blood pressure; GFR, glomerular filtration rate; IQR, interquartile range; SBP, systolic blood pressure.

^a^ Calculated using the Wilcoxon rank sum test for continuous variables and the Cochran-Armitage test for binary variables.

^b^ Defined as urine protein to creatinine levels of at least 2 mg/mg.

^c^ Defined as the other BP (ie, diastolic for the systolic categories and systolic for the diastolic categories).

**Table 2.** Demographic and Clinical Characteristics by Baseline SBP and DBP Percentiles for 264 Participants With Glomerular Disease

Baseline Characteristic	Baseline SBP Percentile				Baseline DBP Percentile			
	<50th (n = 116)	50th to <90th (n = 95)	≥90th (n = 53)	*P* Value^a^	<50th (n = 106)	50th to <90th (n = 118)	≥90th (n = 40)	*P* Value^a^
Age, median (IQR), y	14 (12-16)	13 (10-16)	14 (10-16)	.40	14 (12-16)	14 (11-16)	11 (8-15)	.02
Male, No. (%)	56 (48.4)	56 (58.9)	28 (52.8)	.38	53 (50.0)	68 (57.6)	19 (47.5)	.86
Black race, No. (%)	17 (14.7)	31 (32.6)	22 (41.52)	<.001	20 (18.9)	36 (30.5)	14 (35.0)	.02
Height percentile, median (IQR)	44 (22-73)	43 (11-78)	36 (8-60)	.23	52 (26-80)	33 (12-66)	32 (5-66)	.01
BMI percentile, median (IQR)	70 (42-89)	85 (63-97)	90 (66-98)	<.001	83 (49-96)	80 (48-95)	84 (62-94)	.88
≥85th Percentile for BMI, No. (%)	38 (32.8)	47 (49.5)	33 (62.3)	<.001	51 (48.1)	49 (41.5)	18 (45.0)	.54
GFR, median (IQR), mL/min/1.73 m^2^	64 (50-82)	60 (45-73)	46 (34-62)	<.001	63 (53-78)	61 (48-79)	37 (30-58)	<.001
Ratio of urine protein to creatinine level, median (IQR), mg/mg	0.38 (0.16-1.13)	0.77 (0.21-1.95)	2.76 (0.54-5.17)	<.001	0.39 (0.19-1.07)	0.74 (0.21-2.05)	4.11 (0.89-5.66)	<.001
Nephrotic range proteinuria, No. (%)^b^	15 (12.9)	21 (22.1)	29 (54.7)	<.001	11 (10.4)	30 (25.4)	24 (60.0)	<.001
Matched BP percentile, median (IQR)^c^	40 (22-59)	73 (51-87)	90 (66-98)	<.001	26 (11-55)	64 (44-85)	94 (83-98)	<.001
Antihypertensive use, No. (%)	109 (94.0)	86 (90.5)	47 (88.7)	.22	101 (95.3)	106 (89.8)	35 (87.5)	.08
ACEi/ARB use, No. (%)	106 (91.4)	76 (80.0)	33 (62.3)	<.001	93 (87.7)	96 (81.4)	26 (65.0)	.003
Immunosuppressant use, No. (%)	43 (37.1)	45 (47.4)	30 (56.6)	.01	46 (43.4)	52 (44.1)	20 (50.0)	.54

Abbreviations: ACEi, angiotensin converting enzyme inhibitor; ARB, angiotensin receptor blocker; BMI, body mass index (calculated as weight in kilograms divided by height in meters squared); DBP, diastolic blood pressure; GFR, glomerular filtration rate; IQR, interquartile range; SBP, systolic blood pressure.

^a^ Calculated using the Wilcoxon rank sum test for continuous variables and the Cochran-Armitage test for binary variables.

^b^ Defined as urine protein to creatinine levels of at least 2 mg/mg.

^c^ Defined as the other BP (ie, diastolic for the systolic categories and systolic for the diastolic categories).

Among those with glomerular CKD (Table 2), systolic BP of at least the 90th percentile was associated with black race (22 of 53 [41.5%]), higher BMI (median, 90th [IQR, 66th-98th] percentile), lower GFR (median, 46 [IQR, 34-62] mL/min/1.73 m^2^), nephrotic proteinuria (≥2 mg of protein/mg of creatinine) (29 of 53 [54.7%]), higher diastolic BP (median, 90th [IQR, 66th-98th] percentile), less ACEi/ARB use (33 of 53 [62.3%]), and greater immunosuppressant use (30 of 53 [56.6%]). Diastolic BP of at least the 90th percentile in children with glomerular disease was associated with younger age (median, 11 [IQR, 8-15] years), black race (14 of 40 [35.0%]), lower height percentiles (median, 32nd [IQR, 5th-66th] percentiles), lower GFR (median, 37 [IQR, 3-58] mL/min/1.73 m^2^), greater urinary protein excretion (median, 4.11 [IQR, 0.89-5.66] mg/mg), higher systolic BP (median, 94th [IQR, 83rd-98th] percentile), and less ACEi/ARB medication use (26 of 40 [65.0%]). A total of 29 of 95 participants (30.5%) had systolic BP from the 50th to less than 90th percentiles, and 26 of 53 (49.1%) had systolic BP of at least the 90th percentile at all of their study visits; 32 of 118 (27.1%) and 23 of 40 (57.5%), respectively, remained in those same diastolic BP categories. In the reference groups (<50th percentile for systolic and diastolic BP), 52 of 116 (44.8%) and 40 of 106 (37.7%), respectively, remained in those categories. Overall, 107 participants (40.5%) were consistently in 1 of 3 groups for systolic BP, and 95 (36.0%) were consistently in 1 of the 3 groups for diastolic BP.

**Figure 1.**
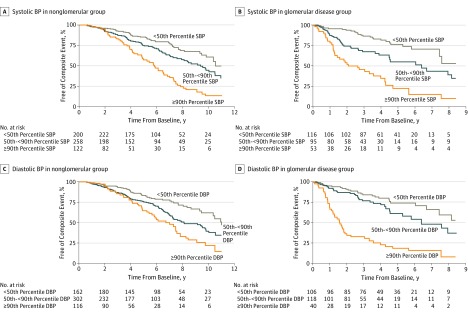
Unadjusted Survival Curves of Progression to the Composite Renal Outcome by Time-Varying Blood Pressure (BP) Percentile Categories The reference group includes those with BP of less than the 50th percentile compared with groups with BP from the 50th to less than 90th percentiles and at least 90th percentile for systolic BP (SBP) and diastolic BP (DBP).

Figure 1 presents nonparametric survival functions for the composite event with time-varying BP percentiles as the exposure, stratified by nonglomerular and glomerular CKD. For systolic BP, (Figure 1A and B), higher BP categories had faster times to the composite renal outcome regardless of CKD etiology. Similar curves were observed for diastolic BP percentiles categories (Figure 1C and D).

**Figure 2.**
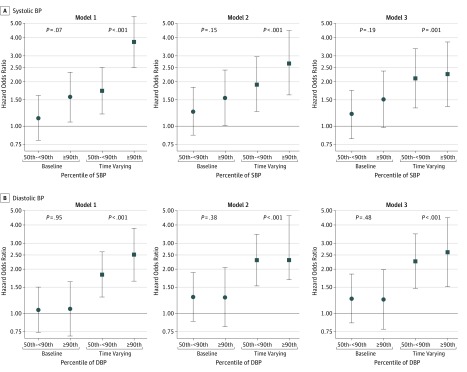
Hazard Odds Ratios for Development of the Composite Renal Outcome Among Participants With Nonglomerular Disease Circles represent hazard odds ratios from baseline blood pressure (BP) models; squares, hazard odds ratios from time-varying BP models. Model 1 is unadjusted; model 2, adjusted for age, sex, black race, antihypertensive use, estimated glomerular filtration rate, and body mass index *z* score; and model 3, adjusted for all covariates in model 2 plus proteinuria. DBP indicates diastolic BP; SBP, systolic BP. Error bars indicate 95% CIs.

**Figure 3.**
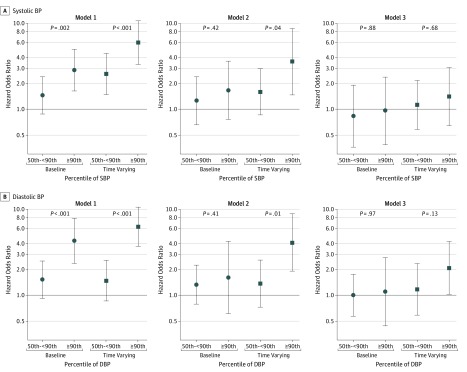
Hazard Odds Ratios for Development of the Composite Renal Outcome Among Participants With Glomerular Disease Circles represent hazard odds ratios from baseline blood pressure (BP) models; squares, hazard odds ratios from time-varying BP models. Model 1 is unadjusted; model 2, adjusted for age, sex, black race, antihypertensive use, estimated glomerular filtration rate, body mass index *z* score, and immunosuppressant use; and model 3, adjusted for all covariates in model 2 plus proteinuria. DBP indicates diastolic BP; SBP, systolic BP. Error bars indicate 95% CIs.

Figure 2 and Figure 3 present the estimated HORs and corresponding 95% CIs to describe the risk of the composite renal outcome associated with higher BP percentiles, stratified by nonglomerular and glomerular causes, respectively (eTable 3 and eTable 4 in the Supplement). Figure 2A and Figure 3A display the association with systolic BP, and Figure 2B and Figure 3B show diastolic BP. Among participants with nonglomerular disease, baseline systolic BP in the 50th to less than 90th percentiles and 90th percentile or greater were associated with an elevated unadjusted HOR for the development of the composite renal outcome compared with participants with systolic BP of less than the 50th percentile (HOR for 50th to <90th percentiles, 1.13 [95% CI, 0.80-1.61]; HOR for ≥90th percentile, 1.58 [95% CI, 1.07-2.32]; *P* = .07 for trend). When time-varying BP was incorporated, the unadjusted HORs were substantially higher (HOR for 50th to <90th percentiles, 1.74 [95% CI, 1.21-2.49]; HOR for ≥90th percentile, 3.75 [95% CI, 2.53-5.57]; *P* < .001 for trend). In the weighted model, higher time-varying systolic BPs were associated with increased risk of the composite renal outcome (adjusted HOR for 50th to <90th percentiles, 1.92 [95% CI, 1.26-2.95]; HOR for ≥90th percentile, 2.67 [95% CI, 1.63-4.45]; *P* < .001 for trend); these were substantially higher than the models with baseline BP as the exposure and adjustment for baseline covariates. Last, in fully adjusted models that included proteinuria, the association of time-varying elevated systolic BP with the composite renal outcome was slightly attenuated but was statistically significant (adjusted HOR for 50th to <90th percentiles, 2.11 [95% CI, 1.33-3.35]; HOR for ≥90th percentile, 2.25 [95% CI, 1.36-3.72]; *P* = .001 for trend) and substantially higher than adjusted baseline models (model 3, adjusted HOR for 50th to <90th percentiles, 1.20 [95% CI, 0.82-1.74]; HOR for ≥90th percentile, 1.52 [95% CI, 1.52-2.36]; *P* = .19 for trend). The same phenomenon was observed when diastolic BP was used as the exposure, with time-varying diastolic BP percentiles being much more strongly associated than baseline-only diastolic BP percentiles.

A similar phenomenon was observed among those with glomerular CKD. Specifically, in unadjusted models, for baseline only SBP of the 90th percentile or greater compared with less than the 50th percentile, the HOR was 2.85 (95% CI, 1.64-4.94), whereas the corresponding estimate for time-varying SBP was substantially higher (HOR, 5.96 [95% CI, 3.37-10.54]). When including proteinuria, the associations with BP were substantially attenuated for baseline BP (≥90th percentile compared with <50th percentile adjusted HOR for baseline, 0.97 [95% CI, 0.39-2.36]) and time-varying BP (≥90th percentile compared with <50th percentile adjusted HOR for time-varying, 1.41 [95% CI, 0.65-3.03]), indicating the importance of proteinuria in this group.

These associations were consistent using diastolic BP as the exposure among glomerular CKD; baseline BP had substantially attenuated HORs compared with the incorporation of time-varying BP. However, the association of time-varying diastolic BP of at least the 90th percentile with the composite renal outcome remained significant after adjustment including proteinuria (adjusted HOR, 2.08; 95% CI, 1.03-4.21) but was null for those with time-varying diastolic BP from the 50th to less than 90th percentiles (adjusted HOR, 1.18; 95% CI, 0.60-2.32).

## Discussion

Time-varying systolic and diastolic BP measurements were associated with an increased risk of CKD progression compared with baseline BP in a study cohort of children with CKD, with a differential association dependent on CKD etiology. Risk associated with development of the composite outcome was 3-fold higher for time-varying systolic BP from the 50th to less than 90th percentiles and the 90th or greater percentile than for baseline systolic BP in participants with nonglomerular CKD, with a similar 3-fold rise in risk with time-varying diastolic BP from the 50th to less than 90th percentiles and the 90th or greater percentile compared with baseline diastolic BP of less than the 50th percentile. In participants with glomerular CKD, only time-varying diastolic BP of the 90th or greater percentile was associated with a 2-fold increased risk of the composite outcome after adjustment for proteinuria. We believe our findings confirm that time-varying BP more accurately represents hypertension management over time in pediatric CKD and demonstrates a significantly higher increased risk for CKD progression when hypertension remains uncontrolled over time.

The association between hypertension at presentation and progression of CKD has been well described in adult and pediatric cohorts.^4,20,21,22^ Our data appear to extend these findings and reinforce the importance of achieving and maintaining systolic BP at least less than the 90th percentile. Moreover, the associations among hypertension, proteinuria, and CKD progression are intertwined and codependent,^14^ as shown in our analyses adjusted for proteinuria. In children with glomerular CKD, adjustment for proteinuria negated almost all associations with baseline or time-varying BP. In contrast, adjustment for proteinuria in children with nonglomerular CKD only attenuated the magnitude of association, but the risk of CKD progression remained significant. The differential association of proteinuria may be related to the underlying pathological processes, because proteinuria in glomerular CKD is often representative of a more active disease process, whereas proteinuria in nonglomerular CKD may have a proportionately larger tubular element, may be structurally related, and may be less likely to be associated with disease activity.^23^ Nephrotic-range proteinuria was highly prevalent in the children with glomerular diagnoses in this cohort, which may account for the loss of statistical significance in the proteinuria-adjusted models among children with glomerular CKD. Without a larger sample size, we are unable to discern the independent associations of proteinuria and hypertension with CKD progression. Nonetheless, our findings suggest that BP and proteinuria control should be optimized in children with glomerular CKD.

### Strengths and Limitations

This study has several strengths. The CKID cohort represents one of the largest pediatric cohorts of CKD, with a large number of high-risk participants with complete follow-up data collected prospectively during several years. The composite outcome was clearly defined, with a central laboratory assessment of GFR. Other study strengths include a standardized BP measurement protocol,^2^ which follows recommendations for BP measurement in children.^24^ Analytically, we used epidemiological tools to address potential time-varying confounding owing to prior exposures as well as biases owing to informative dropout, and incorporated time-varying BP to provide estimates of the association between BP and the composite outcome that are more valid. Because this approach used much more information than baseline values alone, our results present a comprehensive assessment of the risk of elevated BP in children with CKD.

However, measuring BP in children carries an inherent difficulty in obtaining accurate noninvasive measurements during a single visit, even when measurements are repeated within that visit. There is intrinsic variability within individual health care clinicians and between measurement techniques.^25^ Although office BP measurement is the most common method of assessment, use of office BP readings to identify and monitor hypertension has limitations.^24,26,27^ Accurate measurement of BP in younger children often has practical difficulties, such as poor patient cooperation and more frequent use of automated BP machines. Despite these issues, auscultation is widely accepted and endorsed by consensus organizations. To mitigate this issue, we used the mean of 3 BP measurements taken at least 30 seconds apart and recommend not relying on a single BP measurement in clinical practice. Repeated auscultated BP measurement should be conducted to provide more accurate estimate of risk for CKD progression.^24,28^ In this analysis, BP percentiles were classified into 3 groups, and we acknowledge that misclassification was possible, although it likely affected the time-fixed models more than analyses that incorporated time-varying data.

A prior analysis within a subset of the CKID cohort confirmed the prevalence of masked hypertension (ie, normal office BP but elevated BP on ambulatory monitoring) varying from 36% to 49% of the cohort over time.^29^ This study design did not identify children with masked hypertension because repeated ambulatory blood pressure monitoring was not available in the entire cohort. Based on the present study design, children with masked hypertension in the BP groups of less than the 50th percentile or between the 50th and 90th percentiles may be at a greater risk of CKD progression; thus, our results may provide a conservative estimate of risk. Owing to limited power, we could not conduct a more granular evaluation of percentile of BP (eg, 75th vs 95th vs 99th percentiles). An additional limitation is the uncertainty regarding medication adherence. This information was limited because this was an observational rather than interventional study. Furthermore, these models assume constant elevated risk within BP categories, yet risk may vary within each category. For example, those with a BP in the 97th percentile may have a higher risk than those with a BP in the 91st percentile, although our designated BP categories assumed similar risk. Although the inverse probability weights were most efficiently constructed according to these clinically meaningful categories, we recognize that there may be heterogeneous risk within groups.

## Conclusions

In this cohort study among children with CKD, elevated time-varying BP was associated with 2 to 3 times greater risk of progressive CKD, regardless of disease etiology, and was more strongly associated with risk than baseline BP. Among participants with glomerular CKD, proteinuria was strongly associated with progressive CKD and attenuated the BP association. These findings suggest that analyses that only use baseline BP likely underestimate the true association of elevated BP with CKD progression. We believe this study emphasizes the importance of optimizing BP control in children to slow CKD progression.

## Notes

Supplement. **eTable 1. **Number of Study Visits and Duration of Follow-up Stratified by Diagnosis **eTable 2. **Composite Renal Outcome Events Stratified by Etiology **eTable 3. **Hazard Odds Ratio for Development of Composite Outcome of ESRD Among 580 Participants With Nonglomerular Disease **eTable 4. **Hazard Odds Ratio for Development of Composite Outcome of ESRD Among 264 Participants With Glomerular Disease
