# Supplementary material for: Association of Time-Varying Blood Pressure With Chronic Kidney Disease Progression in Children
Source: JAMA Netw Open. 2020 Feb 14;3(2):e1921213. doi: 10.1001/jamanetworkopen.2019.21213 (PMC7236873; doi:10.1001/jamanetworkopen.2019.21213)
Supplement: Supplement. — eTable 1. Number of Study Visits and Duration of Follow-up Stratified by Diagnosis eTable 2. Composite Renal Outcome Events Stratified by Etiology eTable 3. Hazard Odds Ratio for Development of Composite Outcome of ESRD Among 580 Participants With Nonglomerular Disease eTable 4. Hazard Odds Ratio for Development of Composite Outcome of ESRD Among 264 Participants With Glomerular Disease [file jamanetwopen-3-e1921213-s001.pdf]

Supplementary Online Content

Reynolds BC, Roem JL, Ng DKS, et al. Association of time-varying blood pressure with chronic kidney disease progression in children. *JAMA Netw Open.* 2020;3(2):e1921213. doi:10.1001/jamanetworkopen.2019.21213

eTable 1. Number of Study Visits and Duration of Follow-up Stratified by Diagnosis

eTable 2. Composite Renal Outcome Events Stratified by Etiology

eTable 3. Hazard Odds Ratio for Development of Composite Outcome of ESRD Among 580 Participants With Nonglomerular Disease

eTable 4. Hazard Odds Ratio for Development of Composite Outcome of ESRD Among 264 Participants With Glomerular Disease

This supplementary material has been provided by the authors to give readers additional information about their work.

© 2020 Reynolds BC et al. *JAMA Network Open*.

eTable 1. Number of Study Visits and Duration of Follow-up Stratified by Diagnosis

	**Non-glomerular** (n= 580 participants, 3195 visits)	**Glomerular** (n= 264 participants, 1046 visits)	**Total** (n= 844 participants, 4241 visits)
**Number of visits**			
1	52 (9%)	44 (17%)	96 (11%)
2-3	97 (17%)	77 (29%)	174 (21%)
4-5	142 (24%)	84 (32%)	226 (27%)
6-7	163 (28%)	36 (13%)	199 (24%)
8-9	62 (11%)	15 (6%)	77 (9%)
10+	64 (11%)	8 (3%)	72 (8%)
**Years follow-up to event or censoring**	5 [3, 7]	3 [2, 5]	4 [2, 6]
**Time between visits (years)**	0.996 [0.824, 1.104]	0.991 [0.662, 1.095]	0.996 [0.800, 1.101]

© 2020 Reynolds BC et al. *JAMA Network Open*.

eTable 2. Composite Renal Outcome Events Stratified by Etiology

Event type	Events for non-glomerular<br>n= 196	Events for glomerular<br>n= 99
Renal replacement therapy (dialysis or transplant)	130 (66%)	58 (59%)
50% decline in GFR	62 (32%)	40 (40%)
GFR < 15 ml/min|1.73m^2	4 (2%)	1 (1%)

© 2020 Reynolds BC et al. *JAMA Network Open*.

eTable 3. Hazard Odds Ratio for Development of Composite Outcome of ESRD Among 580 Participants With Nonglomerular Disease

		Model 1	Model 2	Model 3
**Baseline SBP %ile**	**<50th**	1 (ref)	1 (ref)	1 (ref)
	**50th-90th**	1.13 (0.80, 1.61)	1.25 (0.87, 1.82)	1.20 (0.82, 1.74)
	**≥90th**	**1.58 (1.07, 2.32)**	**1.55 (1.00, 2.40)**	1.52 (0.98, 2.36)
	**P for trend**	0.07	0.15	0.19
**Time-varying SBP %ile**	**<50th**	1 (ref)	1 (ref)	1 (ref)
	**50th-90th**	**1.74 (1.21, 2.49)**	**1.92 (1.26, 2.95)**	**2.11 (1.33, 3.35)**
	**≥90th**	**3.75 (2.53, 5.57)**	**2.67 (1.63, 4.45)**	**2.25 (1.36, 3.72)**
	**P for trend**	**<.001**	**<.001**	**0.001**
**Baseline DBP %ile**	**<50th**	1 (ref)	1 (ref)	1 (ref)
	**50th-90th**	1.05 (0.74, 1.50)	1.29 (0.88, 1.89)	1.25 (0.86, 1.84)
	**≥90th**	1.07 (0.70, 1.63)	1.28 (0.81, 2.04)	1.24 (0.78, 1.97)
	**P for trend**	0.95	0.38	0.48
**Time-varying DBP %ile**	**<50th**	1 (ref)	1 (ref)	1 (ref)
	**50th-90th**	**1.83 (1.29, 2.60)**	**2.30 (1.54, 3.43)**	**2.26 (1.48, 3.45)**
	**≥90th**	**2.51 (1.66, 3.78)**	**2.80 (1.70, 4.59)**	**2.60 (1.52, 4.45)**
	**P for trend**	**<.001**	**<.001**	**<.001**

Model 1: unadjusted model

Model 2: model 1 plus adjustment for GFR, antihypertensive use (ACEi/ARB and non-ACEi/ARB), immunosuppressant use (G only), age, male sex, black race, BMI z-score (6 events and 97 person-visits removed from time-varying models due to missing BMI)

Model 3: model 2 plus adjustment for proteinuria (8 events and 110 person-visits removed from time-varying models due to missing BMI and/or proteinuria)

Bold indicates significance.

Participants contributed 3195 person-visits and 196 composite events.

© 2020 Reynolds BC et al. *JAMA Network Open*.

eTable 4. Hazard Odds Ratio for Development of Composite Outcome of ESRD Among 264 Participants With Glomerular Disease

		Model 1	Model 2	Model 3
**Baseline SBP %ile**	**<50th**	1 (ref)	1 (ref)	1 (ref)
	**50th-90th**	1.45 (0.89, 2.36)	1.26 (0.67, 2.37)	0.84 (0.37, 1.90)
	**≥90th**	**2.85 (1.64, 4.94)**	1.66 (0.77, 3.61)	0.97 (0.39, 2.36)
	**P for trend**	**0.002**	0.42	0.88
**Time-varying SBP %ile**	**<50th**	1 (ref)	1 (ref)	1 (ref)
	**50th-90th**	**2.57 (1.49, 4.43)**	1.60 (0.87, 2.94)	1.13 (0.59, 2.15)
	**≥90th**	**5.96 (3.37, 10.54)**	**3.60 (1.50, 8.66)**	1.41 (0.65, 3.03)
	**P for trend**	**<.001**	**0.04**	0.68
**Baseline DBP %ile**	**<50th**	1 (ref)	1 (ref)	1 (ref)
	**50th-90th**	1.52 (0.93, 2.50)	1.34 (0.80, 2.25)	1.01 (0.58, 1.75)
	**≥90th**	**4.32 (2.38, 7.82)**	1.62 (0.62, 4.25)	1.11 (0.45, 2.73)
	**P for trend**	**<.001**	0.41	0.97
**Time-varying DBP %ile**	**<50th**	1 (ref)	1 (ref)	1 (ref)
	**50th-90th**	1.48 (0.87, 2.54)	1.38 (0.74, 2.57)	1.18 (0.60, 2.32)
	**≥90th**	**6.28 (3.75, 10.53)**	**4.11 (1.92, 8.79)**	**2.08 (1.03, 4.21)**
	**P for trend**	**<.001**	**0.01**	0.13

Model 1: unadjusted model

Model 2: model 1 plus adjustment for GFR, antihypertensive use (ACEi/ARB and non-ACEi/ARB), immunosuppressant use (G only), age, male sex, black race, BMI z-score (4 events and 55 person-visits removed from time-varying models due to missing BMI)

Model 3: model 2 plus adjustment for proteinuria (4 events and 59 person-visits removed from time-varying models due to missing BMI and/or proteinuria)

Bold indicates significance.

Participants contributed 1046 person-visits and 99 events.

© 2020 Reynolds BC et al. *JAMA Network Open*.
